# Phagocytic Uptake of Oxidized Heme Polymer Is Highly Cytotoxic to Macrophages

**DOI:** 10.1371/journal.pone.0103706

**Published:** 2014-07-31

**Authors:** Rohitas Deshmukh, Vishal Trivedi

**Affiliations:** Malaria Research Group, Department of Biotechnology, Indian Institute of Technology-Guwahati, Assam, India; Universidade Federal do Rio de Janeiro, Brazil

## Abstract

Apoptosis in macrophages is responsible for immune-depression and pathological effects during malaria. Phagocytosis of PRBC causes induction of apoptosis in macrophages through release of cytosolic factors from infected cells. Heme polymer or β-hematin causes dose-dependent death of macrophages with LC_50_ of 132 µg/ml and 182 µg/ml respectively. The toxicity of hemin or heme polymer was amplified several folds in the presence of non-toxic concentration of methemoglobin. β-hematin uptake in macrophage through phagocytosis is crucial for enhanced toxicological effects in the presence of methemoglobin. Higher accumulation of β-hematin is observed in macrophages treated with β-hematin along with methemoglobin. Light and scanning electron microscopic observations further confirm accumulation of β-hematin with cellular toxicity. Toxicological potentiation of pro-oxidant molecules toward macrophages depends on generation of H_2_O_2_ and independent to release of free iron from pro-oxidant molecules. Methemoglobin oxidizes β-hematin to form oxidized β-hematin (βH*) through single electron transfer mechanism. Pre-treatment of reaction mixture with spin-trap Phenyl-N-t-butyl-nitrone dose-dependently reverses the β-hematin toxicity, indicates crucial role of βH* generation with the toxicological potentiation. Acridine orange/ethidium bromide staining and DNA fragmentation analysis indicate that macrophage follows an oxidative stress dependent apoptotic pathway to cause death. In summary, current work highlights mutual co-operation between methemoglobin and different pro-oxidant molecules to enhance toxicity towards macrophages. Hence, methemoglobin peroxidase activity can be probed for subduing cellular toxicity of pro-oxidant molecules and it may in-turn make up for host immune response against the malaria parasite.

## Introduction

Malaria caused by *Plasmodium falciparum* completes its life cycle in the vertebrate host human and invertebrate host mosquito [1], [2]. In the vertebrate host, parasite spends a major part of its life-cycle inside red blood cells (RBCs) and depends on hemoglobin for nutrition [3], [4]. Rupture of parasite containing RBCs leads to release a large amount of hemoglobin/methemoglobin, free hemin, haemozoin, malaria toxins, and other uncharacterized metabolic by-products [5], [6], [7]. Hemin, haemozoin, methemoglobin and other iron containing hemoglobin degradation products are pro-oxidant in nature and have potential to cause oxidative damage to the cells and tissues [7], [8], [9], [10], [11], [12]. Exposure of pro-oxidant molecules to the immune cells exhibit a change in their cytokine secretion profile and contributes in inflammation during malaria [11], [12], [13]. Circulatory phagocytes (monocytes) and tissue associated macrophages provide defense from invading pathogens [14], [15]. Macrophages exposed to haemozoin or β-hematin (synthetic haemozoin) is responsible for immune-depression during malaria. Also, it exhibits depression of phagocytosis, inhibition of phagosome/lysosome maturation and disturbance of pro-inflammatory/anti-inflammatory cytokine balance. [16], [17], [18], [19], [20]. Phagocytosis of polymeric haemozoin is responsible for inhibition of phagosome/lysosome fusion through release of free iron [19], [21]. Immune-depression during malaria is also due to the depression of proliferative behavior of peripheral blood mononuclear cells (PBMC) towards antigen, decreased number of T-lymphocytes, circulating phagocytes, neutrophils and macrophages [22], [23], [24], [25]. Inflammation at brain site through aberrant activation of immune cells (T-cells or phagocytes) leads to leaky behavior of endothelial cells. It results in accumulation of parasitized RBCs and pathological complication of brain [26], [27]. Pro-oxidant molecules released during malaria contribute in pathological complications of cerebral malaria, vascular complication and immune-depression following multiple mechanisms, but still it is not conclusive [28], [29], [30].

A comparative analysis of *P.falciparum* infected patients with control healthy volunteers indicates high degree of spontaneous apoptosis in mononuclear phagocytes [31], [32], [33]. In-vitro exposure of *P.falciparum* antigen extract to monocytes induces apoptosis in 87.5% of the subject tested, but the mechanistic details are not clear [33]. RBCs infected with *P.falciparum* express phosphatidyl-serine on their outer surface [34] and phagocytosis of infected RBCs induces macrophage apoptosis following a redox imbalance [19], [35]. The macrophage apoptosis does not depends on phagocytosis whereas cellular factor(s) of RBCs are responsible for oxidative stress mediated induction of apoptosis. Hemoglobin and hemin derived from hemoglobin are the causative agent for macrophage apoptosis [35]. In-vitro exposure of macrophages with methemoglobin causes dose-dependent induction of apoptosis with generation of multiple intracellular reactive oxygen species (ROS) spikes for prolonged period [36]. Methemoglobin has an intrinsic peroxidase activity and oxidizes aromatic and halide substrates to form polymeric products [37], [38], [39]. Methemoglobin accepts free hemin as substrate and forms heme polymer with an identical bonding pattern, crystal structure, and packing to synthetic β-hematin. MetHb-produced heme polymer is a potent pro-inflammatory factor, which stimulates macrophages to secrete large amount of ROS in the external microenvironment [38]. In the current study, we explored mechanistic details of mutual synergistic interaction of different pro-oxidant molecules and associated severe cytotoxic effect towards macrophage. The data supports that the methemoglobin accepts heme polymer as substrate and oxidizes to form oxidized heme polymer to enhance its toxicity. Phagocytosis of crystalline β-hematin is upregulated in the presence of methemoglobin and might be responsible for cyto-toxic effects through oxidative stress mediated apoptosis. Mechanistic details of toxicological potentiation through interaction of pro-oxidant molecules towards immune cells are explored and discussed.

## Materials and Methods

### Chemicals and reagents

Methemoglobin (MetHb), β-hematin (βH), mannitol, N-Acetylcysteine (NAC), Phenyl *N*-t-butylnitrone **(**PBN), catalase, deferoxamine, ethidium bromide, acridine orange, 3-(4,5-dimethylthiazol-2-yl)-2,5-diphenyltetrazolium bromide (MTT), thiobarbituric acid, 1,1′,3,3′ tetraethoxy propane, guanidine hydrochloride, 5,5′ dithiobis (2-nitrobenzoic acid), agarose, In-Vitro Toxicology Assay Kit were purchased from Sigma, St. Louis, MO, USA. Dinitrophenyl hydrazine, ethylacetate, dimethylsulfoxide (DMSO), Triton X-100, hydrogen peroxide and trichloroacetic acid (TCA) were procured from Merck, Germany. Piceatannol was purchased from Calbiochem (San Diego, CA.). Control and anti-TNF-α antibodies were purchased from BD-biosciences. Other reagents and chemicals were of analytical grade purity.

### Cell Culture and Treatments

J774A.1 mouse macrophages were obtained from national cell culture facility, Central Drug Research Institute, Lucknow. Cells were cultured in dulbecco’s modified eagle’s medium (DMEM) supplemented with 10% fetal bovine serum (FBS) and 1% penicillin-streptomycin antibiotic (100 units/ml penicillin and 100 µg/ml streptomycin sulfate) in 100 mm^2^ cell culture dish (Corning, Lowell, MA, USA). 10,000 Cells were seeded over-night, prior to the day of experiments in 0.2 ml complete media. On the day of the experiment, cells were washed 2-times with PBS and treated with MetHb (7.75 µM) or different concentration of β-hematin (0–120 µg/ml) or combination of MetHb (7.75 µM) with different concentration of β-hematin (0–120 µg/ml) for 6 hr at 37°C in serum free media. To test the effect of various antioxidants or spin trap, cells were pre-incubated with NAC (5 mM), Mannitol (5 mM), or PBN (0–300 µM) for 1 hr and treated with different agonists as described before.

### Measurement of Cellular Viability

The cellular viability of macrophages was done by MTT reduction assay as described earlier [38], [40]. Macrophages treated with incomplete media (cell culture medium without serum) were considered as 100% viable and used to express viability of cells in other treatments as described previously. To probe the role of oxidative stress or single electron containing species, cells were pre-incubated for 1 hr with antioxidants or spin trap and then treated with different agonists as described above.

### Lactate Dehydrogenase Assay

LDH activity assay was measured using in-vitro toxicological assay kit. Post treatment, 100 µl of the culture supernant was added in to the reaction mixture and allow to react for 30 mins in dark (covered with aluminum foil). Enzymatic reaction was stopped with 1N HCl and absorbance was read at 490 nm. Total LDH was measured to calculate the percentage of LDH released during different treatments.

### Microscopic observation of macrophages

Cells were treated with different agonists and observed with Nikon Eclipse TS 100 inverted microscope using 20x objective pre and post treatment. Images were captured with a high resolution camera (Nikon Corp., Japan).

### Preparation and fractionation of malaria culture supernatant

*Plasmodium falciparum* 3D7 was grown in RPMI containing 0.5% albumax II and culture supernatant was prepared as described previously [41]. Malaria culture supernatant was fractionated with ammonium sulfate as described [30]. The different fractions contain variable amount of methemoglobin with fraction P2 contains maximum amount of methemoglobin. The level of methemoglobin in each fraction was determined as described previously [42].

### Estimation of β-hematin inside the macrophages

Macrophages were treated with β-hematin (60 µg/ml), MetHb (7.75 µM) or combination of β-hematin (60 µg/ml)/MetHb (7.75 µM) for 6 hr. Post treatment, cells were washed twice with PBS to remove uningested β-hematin. Cells were lysed by adding 0.1% triton-x 100 in PBS and lysate was clarified by centrifugation at 1000 g for 5 min at 4°C. The supernatant was again centrifuged at 15,000 g for 10 min at 4°C to pellet out the β-hematin present in the cytoplasm. Pellet was washed 2 times to remove contaminating species and free hemin. The resulting pellet was dissolved in 2N NaOH and absorbance was measured at 400 nm. Absorbance of β-hematin (60 µg/ml) was taken as 100% to calculate % β-hematin uptake in macrophages.

To study the phagocytosis-mediated β-hematin uptake, macrophages were equiliberated at particular temperature (37°C/10°C) for 30 min and treated with different pro-oxidant molecules as described. β-hematin was extracted and quantitated as described.

### Scanning Electron Microscopy

SEM samples were prepared as described [36] with slight modification. Post-treatment, cells were washed with ice cold PBS followed by fixation in glutaraldehyde/paraformaldehyde solution for 24 hr at 4°C. Fixed cells were washed twice with PBS and kept in humid atmosphere for 1 hr at 37°C. Humid cells were dehydrated with graded ethanol from 50% to 100% in a vacuum environment. Dehydrated samples were coated with gold film in a Polaron sputter coater and examined in LEO 1430VP Scanning Electron Microscope. The instrumental conditions like EHT, magnification, width and signal were 10 kV, 2.5 KX, 15 mm and SE1 respectively.

### Measurement of Oxidative stress indices

Macrophages were treated with different pro-oxidant molecules as described and lipid peroxidation, protein carbonyl and intracellular reduced glutathione (GSH) levels were measured as described previously [37], [43].

### Acridine orange and Ethidium Bromide (AO-EtBr) staining

Apoptotic and dead cells can be differentiated by staining cells with acridine orange (AO) and ethidium bromide (Et-Br) as described [44]. Stained cells were analyzed immediately at room temperature with FACS Calibur using Cell Quest pro software (BD Biosciences, USA). Quadrant statistics was performed to determine healthy, early apoptotic, highly apoptotic and dead cells from the total population. Macrophages treated with serum free media were considered as control.

### DNA fragmentation analysis

5×10^5^ cells were treated with MetHb (7.75 µM), β-hematin (60 µg/ml), or β-hematin (60 µg/ml)/MetHb (7.75 µM) for 6 hr at 37°C in serum free media. Post treatment, cells were washed twice with ice cold PBS and lysed with lysis buffer (100 mM Tris-Cl pH 8.0 containing 2 mM EDTA and 0.8% w/v SDS). Lysate was treated with 2 µl of DNase free RNase A (50 mg/ml) at 37°C for 30 min to remove RNA present in the sample. Proteinase K (10 µl of 20 mg/ml) was added to samples and incubated for 2 hrs at 50°C. Samples were then mixed with 6x loading buffer (NEB, USA) and resolved on 1.8% agarose gel containing ethidium bromide (30 µg) at 50 mA for 4 h at 4°C. Fragments of DNA were visualized under UV-light and images were captured with a Kodak Gel Logic 1500 imaging system.

### Optical spectral studies

In a total volume of 1 ml PBS, β-hematin (60 µg/ml), MetHb (7.75 µM), hydrogen peroxide (5 mM) was incubated for 12 h at 37°C in the absence or presence of different concentration of PBN (0–600 µM). The reaction mixture was centrifuged at 12000 rpm for 10 min to recover β-hematin. The β-hematin pellet was washed twice with PBS and finally resuspended in 1 ml PBS. A UV-visible scan (250–700 nm) of recovered β-hematin was recorded in Cary 100 UV/VIS spectrophotometer at 25°C with quartz cells of 1 cm light-path.

## Results

### Mutual interaction of pro-oxidant molecules is responsible for malaria culture supernatant toxicity towards macrophages

Malaria culture supernatant is a mixture of hemoglobin, methemoglobin, heme, hemozoin (polymeric heme) and malaria toxins [5], [6], [7]. The level of methemoglobin in malaria patients and in the malaria culture supernatant varies from 0–54 µM [30], [45], [46]. As per the estimate, approximately 200 µM haemozoin is found in *P.falciparum* infected patients [47] where as the level of haemozin at the brain site may be upto 100 µM [48]. Initially, we tested the hypothesis that mutual co-operative relationship of methemoglobin with heme polymer is responsible for enhanced toxicity of malaria culture supernatant towards macrophages. Malaria culture supernatant fractionated by ammonium sulfate gives fractions with varying amount of methemoglobin, fraction P2 contains the maximum amount of methemoglobin. Macrophages treated with combination of P2/heme (P2/heme mixture) or heme polymer (P2/heme polymer mixture) for 6 h reduced cellular viability; indicating interaction of methemoglobin (pro-oxidant) with other pro-oxidant molecules (heme or heme polymer) to enhance cellular toxicity (Table 1). These results were further confirmed by release of LDH in the culture supernatant from the damaged cells (Table 1). The toxicological amplification is more pronounced for heme polymer rather than hemin.

**Table 1:** Effect of P2 with other pro-oxidant molecules on macrophage viability and membrane integrity.

Treatments	Survival (%) ± SD	Δ Cellular viability (% ± SD)	% LDH released
Hemin	47.0±7.6	NA	36.3±3.1
Heme polymer	71.0±6.8	NA	68.1±2.6
Fraction P2	96.9±4.2	100	6.90±1.5
Fraction P2+ Hemin	34.7±3.8	70	22.2±6.6
Fraction P2+ Heme polymer	43.1±4.6	60	37.7±3.9

Macrophage are exposed to P2 (90 µg) alone or in combination with other pro-oxidant molecules present in malaria culture supernatant for 6 hrs and viability was determined by MTT assay where as membrane integrity was measured by LDH release assay. Macrophage exposed to hemin (60 µg/ml), heme polymer (40 µg/ml) was used as control. Change in cellular viability after reconstitution was calculated considering viability of P2 exposed macrophage as 100%. NA = “Not applicable”.

**Figure 1:**
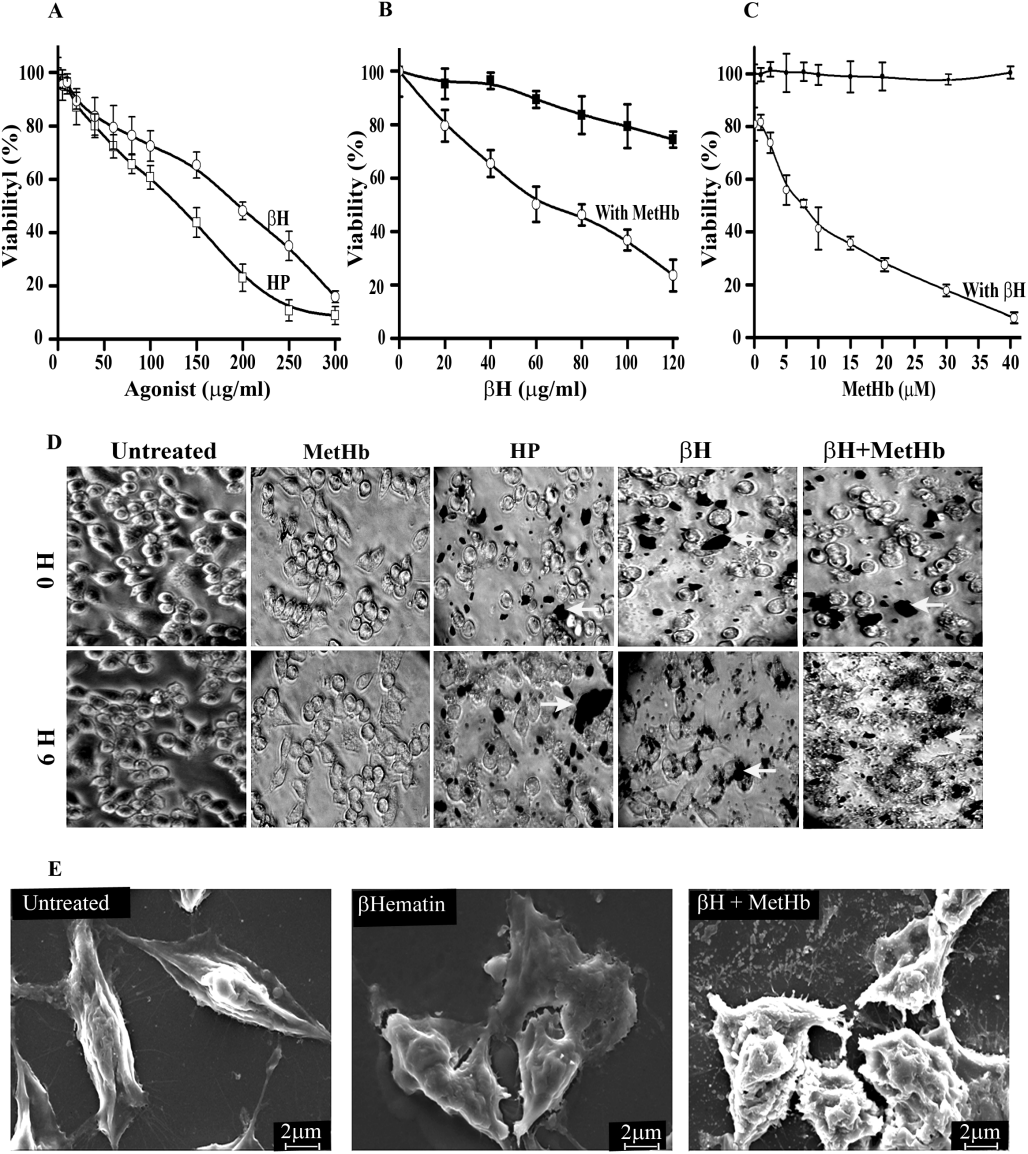
Pro-oxidant molecules co-operate with each other to exhibit enhanced toxicity towards macrophages. (**A**) Evaluation of toxicological potential of heme polymer (HP) and β-hematin (βH) against macrophage J774A.1. Macrophages were treated with different concentration of agonist [heme polymer (HP) or β-hematin (βH)] for 6 hr at 37°C and viability was determined by MTT assay as described in “material and methods”. (**B**) Methemoglobin potentiates the toxicity of β-hematin towards macrophage J774A.1. Macrophages were treated with different concentration of β-hematin (βH) for 6 hr in absence or presence of methemoglobin (7.75 µM) at 37°C and viability was determined by MTT assay as described in “material and methods”. (**C**) β-hematin potentiates the toxicity of methemoglobin towards macrophage J774A.1. Macrophages were treated with different concentration of methemoglobin (0–40 µM) in absence or presence of β-hematin (60 µg/ml) at 37°C and macrophage viability was determined as described in “material and methods”. In panel (A), (B) and (C), macrophages treated with incomplete media were considerd as 100% viable. Data is the mean ± SD of three independent experiments (n = 3) with triplicate measurement. (**D**) Microscopic observation of β-hematin particles and cellular damage in macrophages. Macrophages were either untreated or treated with methemoglobin, heme polymer (HP), β-Hematin (βH) or β-Hematin (60 µg/ml)/methemoglobin (7.75 µM) mixture respectively for 6 hr at 37°C and images of random 10 fields were captured with a 20x objective using an invereted microscope TS100 (Nikon, Japan). β-hematin particles are denoted by arrows in each panel. (**E**) SEM analysis of macrophages. Macrophages were either untreated or treated with β-hematin (60 µg/ml) in the absence or presence of non-toxic concentration of methemoglobin (7.75 µM) for 6 hr at 37°C and a total of 10 different fields were captured using LEO 1430VP Scanning Electron Microscope with the instrument setting as EHT, width and signal were 10 kV, 15 mm and SE1 respectively. A representative image of untreated, treated with β-Hematin (60 µg/ml) or β-hematin (60 µg/ml)/methemoglobin (7.75 µM) mixture respectively (magnification; x2500, scale bar = 2 µm) is given.

Methemoglobin utilizes its pseudoperoxidase activity to oxidize and polymerize aromatic/halide substrates into the polymeric products [37], [38], [39]. MetHb oxidizes hemin to form heme polymer (HP) which has similar crystal packing, bonding pattern and structural features with synthetic β-hematin [38]. The Heme polymer (HP) or β-hematin (βH) exposure to J774A.1 for 6 h dose dependently reduces cellular viability with LC_50_ of 132 µg/ml and 182 µg/ml respectively (Figure 1A). Treating the macrophages with different concentration of βH in the presence of a non-toxic concentration of MetHb (7.75 µM) gives an enhanced level of cellular toxicity with LC_50_ of 58 µg/ml. At the highest concentration of βH (120 µg/ml), methemoglobin enhances its cytotoxicity by 2.75 folds. (Figure 1B). Similarly, heme polymer in the presence of MetHb (7.75 µM) gives a cellular toxicity with LC_50_ of 32 µg/ml. In an earlier study, MetHb exhibits toxicity towards macrophages in 40 h, but it is non-toxic in an earlier time points (<20 hrs) [36]. In the presence of sub-lethal concentration of βH (60 µg/ml), methemoglobin is dose-dependently exhibiting toxicity towards macrophages with LC_50_ of 7.75 µM (Figure 1C). At the highest concentration of MetHb (40 µM), βH enhances its cytotoxicity by 10 folds. Hence, pro-oxidant molecules co-operate synergistically with each other, results in enhanced level of cellular toxicity in an in-vitro toxicity model towards macrophages.

### Methemoglobin exposure causes β-hematin accumulation and morphological deformalities

Macrophage exhibits high level of phagocytotic activity and macrophage population with high phagocytic activity are more susceptible for functional defects and damage from pro-oxidant molecules [49], [50]. Macrophages treated with combination of βH (60 µg/ml)/MetHb (7.75 µM) gives ∼40% more cell associated βH as compared to macrophages treated with βH alone (Table 2). The change in level of cell associated βH is statistically significant (p-value 0.001, βH Vs βH/MetHb). Light microscopic observation of macrophages further confirms the higher level of βH associated with macrophages treated with combination of βH (60 µg/ml)/MetHb (7.75 µM). (Figure 1D, βH crystals are indicated by arrow heads). In addition, careful observation of macrophages treated with βH, or combination of βH (60 µg/ml)/MetHb (7.75 µM) indicates a pronounced cytotoxic effect in βH/MetHb treated cells (Figure 1D). Scanning electron microscopic images of macrophages treated with βH (60 µg/ml)/MetHb (7.75 µM) for 6 h shows an enhanced level of damage to cellular structure, cell shrinkage with severely distorted shape and membrane blebbing (Figure 1E).

**Table 2:** Amount of β-hematin associated with macrophage J774A.1.

Treatments	Total β-hematin (% ± SD)
**Uptake studies at 37**°**C**	
**Methemoglobin**	0.3±0.01
**β-hematin**	10.4±0.6
**β-hematin + Methemoglobin**	14.4±1.1
**Uptake Studies at 10**°**C**	
**Methemoglobin**	0.7±0.2
**β-hematin**	1.0±0.4
**β-hematin + Methemoglobin**	2.1±0.3

β-hematin is a crystalline material, and its uptake inside the macrophages follows phagocytosis mediated internalization and subsequent release into the cytosol through lysis of phagosome membrane [22]. Phagocytosis is an energy dependent process, and it is severely been compromised at subpermisive temperature [51]. Cell associated βH is low (∼1% of total) at 10°C as compared to uptake studies performed at 37°C; it indicates a direct role of phagocytosis in the process (Table 2). To probe the phagocytosis-mediated βH uptake with the toxicity, macrophages were treated with βH (60 µg/ml)/MetHb (7.75 µM) at 10°C for 6 h and cellular viability was determined by MTT assay. Untreated cells incubated at 10°C served as control and used to calculate the change in survival for treated cells. At 37°C in the presence of MetHb (7.75 µM), βH gives ∼50% more toxicity towards macrophages where as at 10°C, the increase in toxicity is only 10% (Table 3). To further probe the phagocytosis-mediated βH uptake with the cytotoxicity of βH/MetHb, macrophages were treated with βH (60 µg/ml)/MetHb (7.75 µM) for 6 h in the absence or presence of cytochalasin D (20 µM) and level of cellular viability was determined by MTT assay. In the presence of cytochalasin D, the cytotoxicity of βH/MetHb is reduced significantly (Table 3). Hence, data in Figure 1 clearly highlights the mutual interaction of pro-oxidant molecules results into the severe damage to cellular structure through higher uptake of βH.

**Table 3:** Toxicity of pro-oxidant molecules towards macrophages at different temperature.

Treatments & Temperature	Survival of macrophage (%)
**Experiment at 37°C**	
**Untreated**	100.0±4.9
**Methemoglobin**	95.0±3.8
**β-hematin**	68.6±2.5
**β-hematin + Methemoglobin**	47.5±0.5
**β-hematin + Methemoglobin + Cytochalasin D (20** µ**M)**	87.1±0.21
**Experiment at 10°C**	
**Untreated**	100.0±15.4
**Methemoglobin**	94.0±11.9
**β-hematin**	74.0±1.4
**β-hematin + Methemoglobin**	67.0±3.3

Macrophages are either untreated or treated with Methemoglobin, β-hematin or combination of β-hemain/Methemoglobin mixture at 37°C or subpermissive temperature (10°C) for 6 hr and the macrophage survival was measured by MTT assay. The experiment is performed in triplicate and values presented were the mean ± SD of four different experiments (n = 4). The MTT absorbance (0.37±0.05) of untreated cells is considered as 100% and used to express the survival of macrophages in other conditions.

### β-hematin accumulation results in enhanced level of Oxidative stress in macrophages

**Figure 2:**
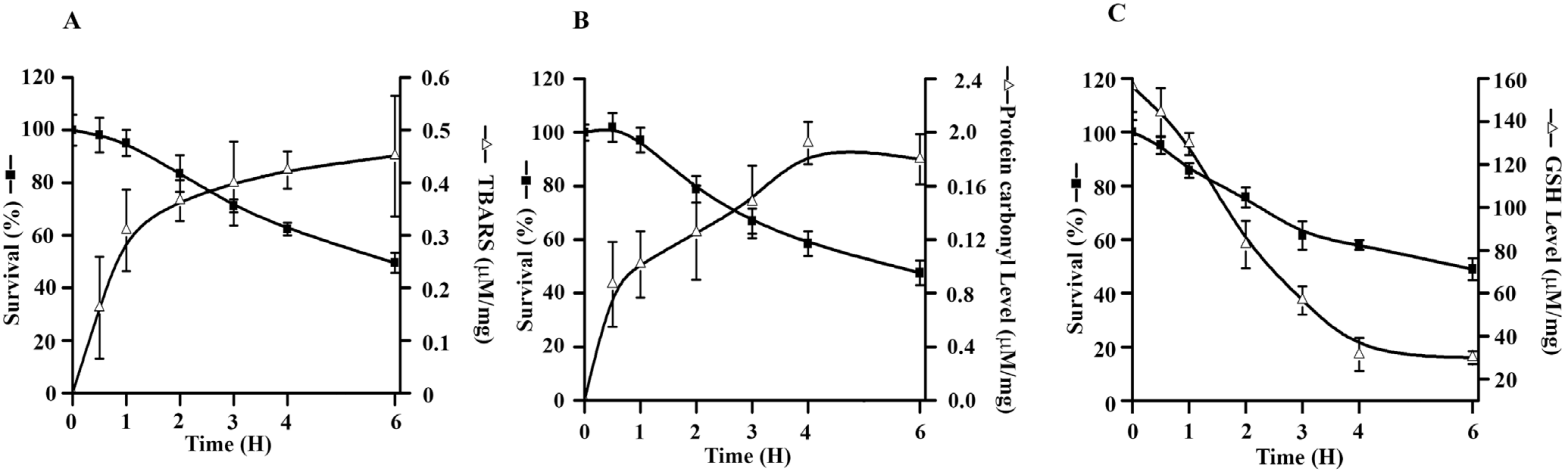
Determination of intracellular oxidative stress indices within macrophages. (**A–C**) Level of oxidative stress indices in macrophages treated with combination of β-hematin (60 µg/ml)/methemoglobin (7.75 µM) over the course of time. Macrophages were either untreated or treated with the combination of β-hematin (60 µg/ml)/MetHb (7.75 µM) for different time points (0–6 hr) and (**A**) lipid peroxidation (**B**) protein carbonyl and (**C**) reduced glutathione is measured and expressed as mM/mg of cell lysate. Data is the mean ± SD of three independent experiments (n = 3) with triplicate measurement. Cellular viability is measured to correlate the change in oxidative stress with the viability of treated cells. The correlation factor (r^2^) for change in viability Vs lipid peroxidation is 0.97, change in viability Vs protein carbonyl is 0.86 and change in viability Vs GSH level is 0.97.

Pro-oxidant molecules disturb cellular physiology and cause development of oxidative stress through inhibition of cellular antioxidant machinery. Methemoglobin exposure produces multiple ROS spikes (periodic increase and decrease of intracellular ROS level) within the macrophage cytosol to exhibit toxicological effects [36]. In contrast, macrophage treated with combination of β-hematin (60 µg/ml)/MetHb (7.75 µM) did not give multiple ROS spike pattern (data not shown). Development of oxidative stress leads to the protein damage, oxidation of membrane lipids, and depletion of antioxidants (GSH) inside the macrophages. As compared to untreated cells, MetHb, βH or combination of β-hematin/MetHb-treated cells shows 5.5%, 20.5% and 61% increase in lipid peroxidation level respectively (Table 4). Similarly, treated cells show 32% increase in protein carbonyl level, whereas MetHb (7.75 µM) or β-hematin (60 µg/ml) treated cells show only 3.4% and 20% respectively (Table 4). An increase in lipid peroxidation or protein carbonyl level is due to reduction of GSH level inside the cell. Cells stimulated with MetHb (7.75 µM), β-hematin (60 µg/ml) or combination of β-hematin (60 µg/ml)/MetHb (7.75 µM) give decrease in GSH level inside the cell by 29%, 34% and 94% respectively (Table 4). The time dependent exposure of macrophages to combination of β-hematin (60 µg/ml)/MetHb (7.75 µM) gives a dose-dependent increase in lipid peroxidation, protein carbonyl, and decrease of GSH (Figure 2A–C). A strong correlation (∼0.97) between macrophage viability and change in oxidative stress indices was observed. The time-dependent progression indicates that the oxidative stress developed first followed by induction of apoptosis mediated cellular death (Figure 2A–C). The data presented clearly indicates a synergistic relationship between MetHb and βH to develop oxidative stress inside the macrophages, and probably be responsible for cellular damage and death.

**Table 4:** Change in level of lipid peroxidation, protein carbonyl and GSH in J774A.1.

Treatments	Increase in lipidperoxidationlevel (%)	Increase in protein carbonyllevel (%)	Decrease in GSHlevel (%)
MetHb	5.6±3.4	3.5±1.4	29.2±1.8
BH	20.6±3.7	18.3±2.8	34.5±6.9
BH + MetHb	62.0±2.0	31.2±4.0	93.9±4.8

Macrophages were treated with different agonist for 6 h and lipid peroxidation, protein carbonyl and GSH level were measured as described under “material and method section”. Untreated cells were taken as control. The values presented were the mean ± SD of three different experiments (n = 3).

### Enhanced Oxidative Stress is responsible for macrophage death

**Figure 3:**
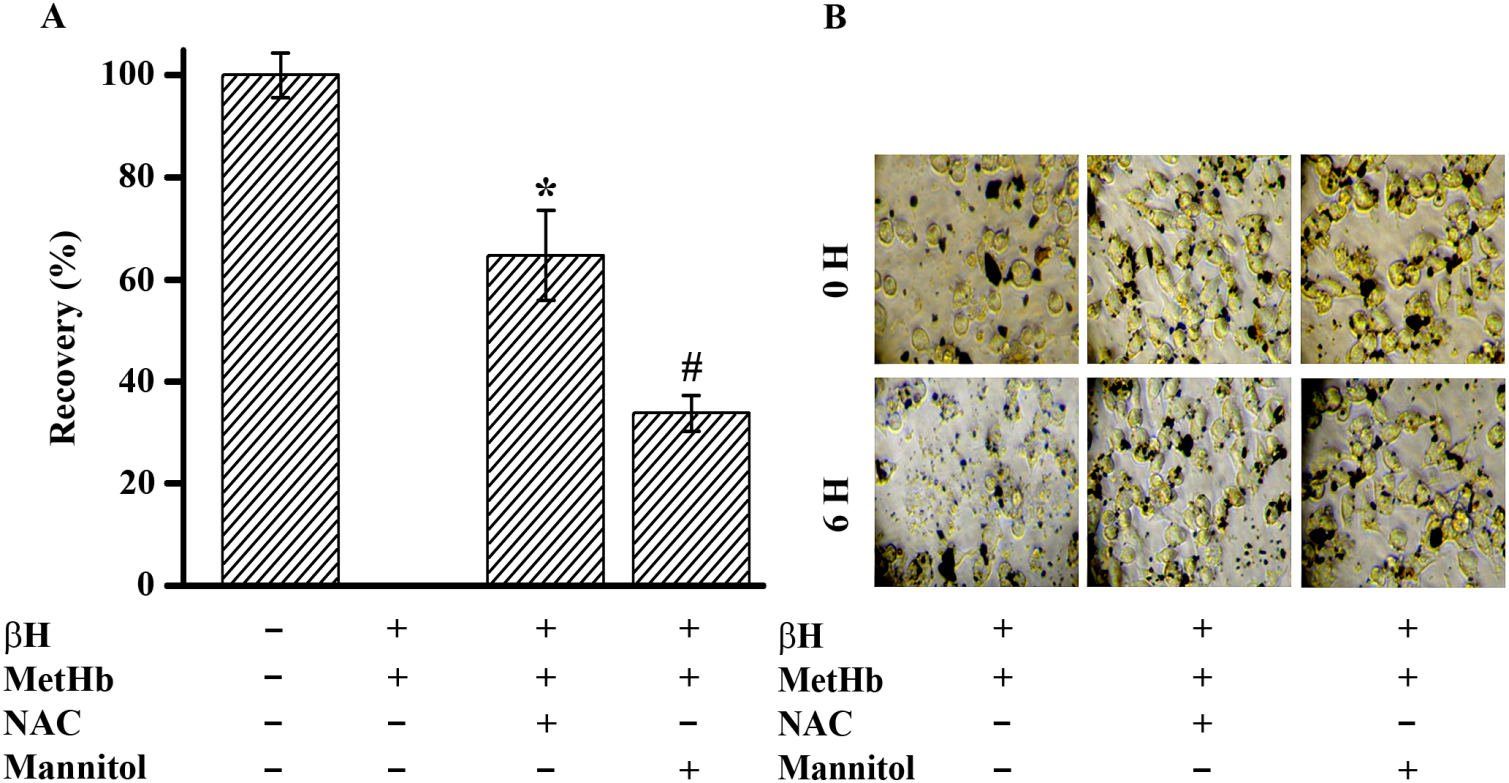
Oxidative stress is required for interaction of different pro-oxidant molecules (β-hematin/methemoglobin) to exhibit enhanced toxicity in macrophages. (**A**) Removal of oxidative stress through anti-oxidant treatement provides recovery in macrophages from the toxicity of β-hematin/methemoglobin mixture. Macrophages were either untreated or treated with β-hematin (60 µg/ml)/methemoglobin (7.75 µM) for 6 hr at 37°C in the absence or presence of NAC (5 mM) and mannitol (5 mM) respectively. Cell viability was measured by MTT assay as described in “material and methods”. Macrophage treated with incomplete media was considered as 100% viable. Macrophage treated with combination of β-hematin (60 µg/ml)/methemoglobin (7.75 µM) was considered as 0% recovery and the cellular viability in the presence of NAC (5 mM) or mannitol (5 mM) was calculated and expressed as % recovery ±SD. Data is the mean ± SD of three independent experiments (n = 3) with triplicate measurement. The pairwise results were analyzed with Anova & Student t-test and it was considered statistically significant with *P<0.001, #P<0.001. (**B**) Light microscopic observation of macrophages treated in (A) with 20x objective to detect cellular morphology at 0 hr and 6 hr.

To verify the oxidative stress mediated toxicity in treated macrophages, ROS was reduced by antioxidants N-acetyl cysteine (NAC) and mannitol. Pre-incubation of treated cells with NAC (5 mM) or mannitol (5 mM) for 1 h shows a significant recovery of death from treated cells in MTT assay. NAC and mannitol have reversed the toxic effects of βH/MetHb by 73% and 38% respectively (Figure 3A). Light microscopic observation of macrophages treated with βH/MetHb in the presence of antioxidants (NAC or mannitol) further confirms the reversal of cellular damages (Figure 3B). After 6 h of treatment, macrophage shows cellular damage phenotype whereas NAC or mannitol pre-incubated cells were found healthy with normal morphology (Figure 3B). NAC is known to increase the intracellular thiol pool (GSH level) to reduce the cellular oxidative stress but measurement of GSH indicates no restoration of GSH level inside the cell (data not shown). In addition, NAC fuctions as an extracellular agent preventing membrane damage. It highlights the role of oxidative stress with the cyto-toxic potentials of βH/MetHb but probably oxidative stress does not regulate βH uptake inside the macrophages.

### Extracellular H_2_O_2_ generation is crucial for toxicological effects of βH/MetHb combination toward macrophages

**Figure 4:**
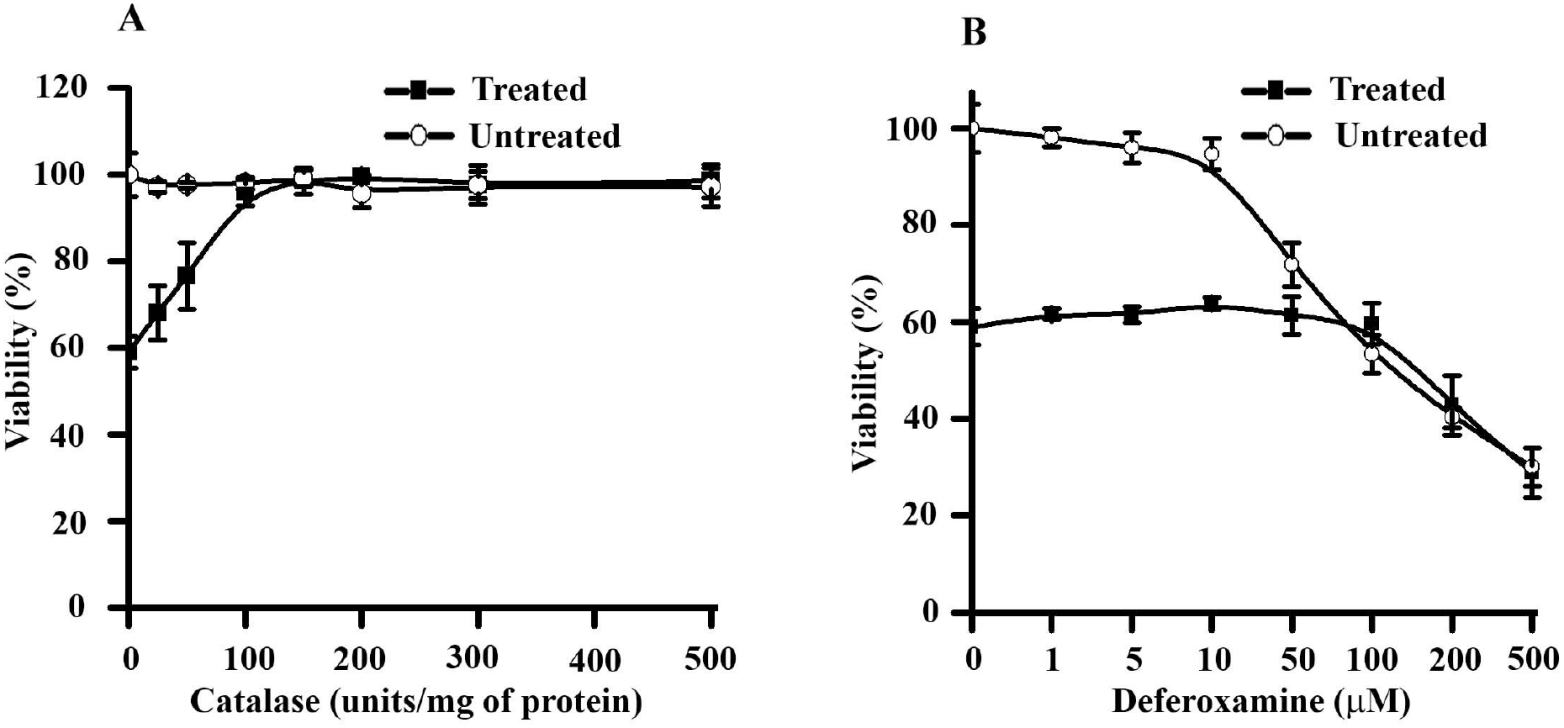
Extracellular H_2_O_2_ Generation is responsible for methemoglobin mediated βH toxicological potentiation towards macrophages. (**A**) Removal of extracellular H_2_O_2_ gives recovery from cytotoxic effects of β-hematin towards macrophages. Macrophages were pre-incubated with different amount of catalase (0–500 U) and either remains untreated or treated with combination of β-hematin (60 µg/ml)/methemoglobin (7.75 µM) for 6 hr at 37°C. Macrophage viability was determined by MTT assay as described in “material and methods” and expressed as % viability ± SD. (**B**) Scavenging free iron has no effect on reversal of cytotoxic effects of β-hematin towards macrophages. Macrophages were pre-incubated with different amount of deferoxamine (0–500 µM) and either remains untreated or treated with combination of β-hematin (60 µg/ml)/methemoglobin (7.75 µM) for 6 hr at 37°C. Macrophage viability was determined by MTT assay as described in “material and methods” and expressed as % viability ± SD. Data is the mean ± SD of three independent experiments (n = 3) with triplicate measurement.

β-hematin has iron co-ordinately associated with the porphyrin ring system [52]. MetHb is a metallo-protein and contains iron (Fe^3+^) in the bound hemin [8]. Release of free iron from the βH or MetHb leads to the production of hydrogen peroxide (H_2_O_2_) and is known to be associated with the toxicity of both pro-oxidant molecules [53], [54]. To probe the role of extracellular hydrogen peroxide generation with toxicological phenotype, H_2_O_2_ level was reduced by catalase and macrophage viability was studied by MTT assay. Treated cells pre-incubated with catalase (0–500 units) show dose-dependent reversal of cytotoxic effects of βH/MetHb (Figure 4A). Macrophage treated with catalase (0–500 IU) alone has no stimulatory or pro-supportive growth effects; it clearly ruled out any such possibility with the observed reversal in cytotoxicity (Figure 4A). To test the possibility of released free iron as a source of H_2_O_2_, cells were treated with combination of βH/MetHb in the presence of deferoxamine (0–500 µM) to chelate free iron. Interestingly, chelating iron has no effect on the reversal of βH/MetHb mediated cytotoxicity towards macrophages (Figure 4B). Macrophage treated with deferoxamine (0–500 µM) alone has no cytotoxic effects up to 10 µM but exerts cytotoxicity beyond this in dose-dependent manner (data not shown). The non-toxic concentration of deferoxamine (0–10 µM) is used to provide recovery in the cellular system through chelation of free iron released from hemo-proteins [55]. Above results clearly indicate pivotal role of extracellular H_2_O_2_ generation but the release of free iron from βH/MetHb mixture has no role in eliciting the cyto-toxicity towards the macrophages.

### Extracellular H_2_O_2_ assists MetHb to oxidize β-hematin via single electron transfer mechanism

**Figure 5:**
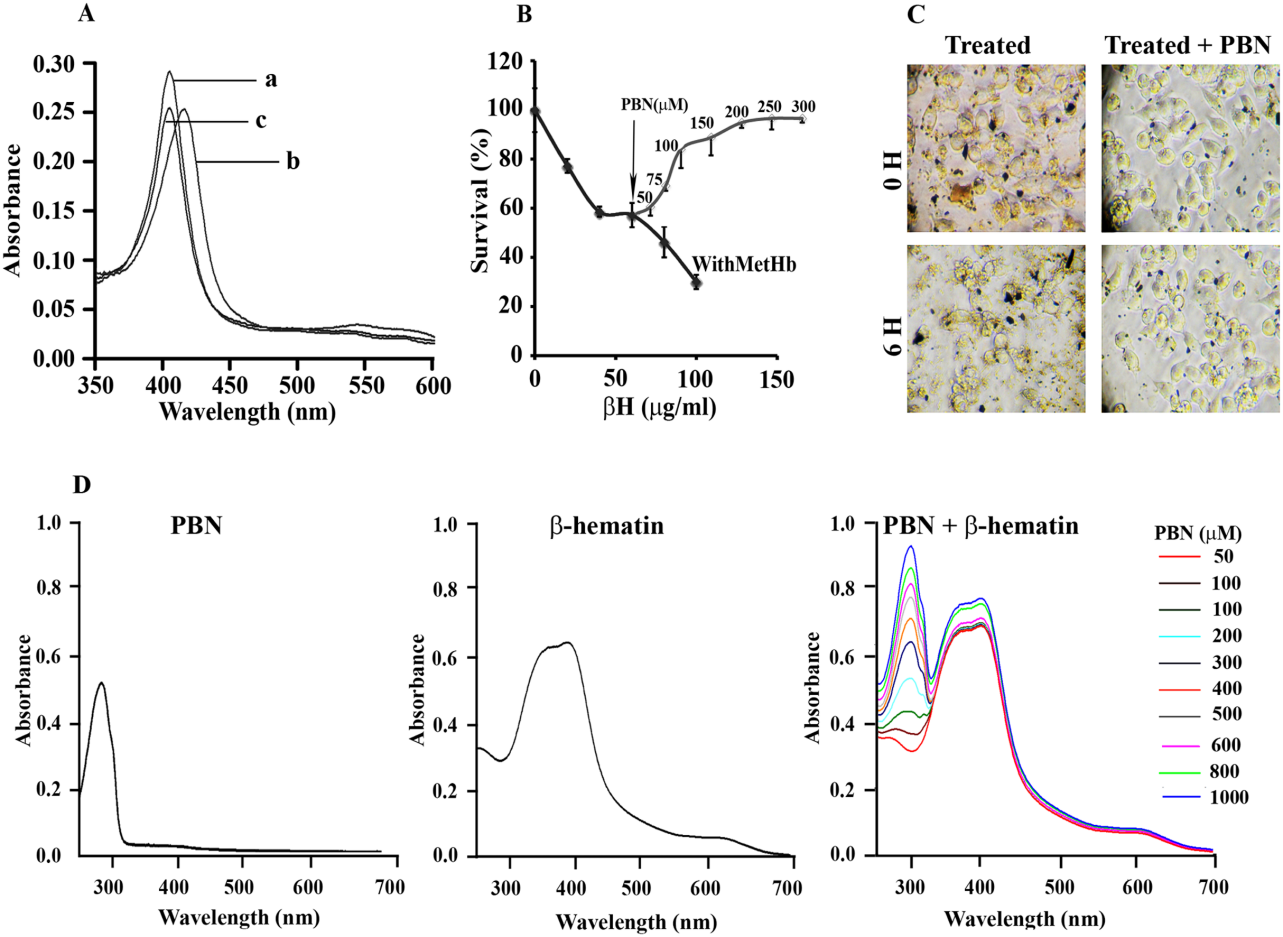
MetHb and β-hematin interaction generate single electron containing species (β-hematin*) to exhibit cyto-toxicity towards macrophages. (**A**) Optical spectra of β-hematin oxidation by methemoglobin. Soret spectra were recorded in 100 mM Tris-HCl buffer, pH 7.4, in a total volume of 0.8 ml. Soret spectrum (a) of MetHb (1 µM); (b) a + H_2_O_2_ (100 µM); (c) b + β-hematin (10 µM). Equal concentration of β-hematin (10 µM) was added in the reference cuvette to correct the absorbance in soret region. (**B**) Scavenging single electron containing species (β-hematin*) restores cellular viability of macrophages. Cells were treated with different concentration of β-hematin (0–150 µg/ml)/methemoglobin (7.75 µM) mixture in the presence of different concentration of PBN (50–300 µM) or remained untreated. Cellular viability was determined by MTT assay as described in “material and methods”. Cells treated with incomplete media was considered as 100% viable. Data is the mean ± SD of three independent experiments (n = 3) with triplicate measurement. (**C**) Light microscopic observation of macrophages treated in (B) with 20x objective to detect cellular morphology at 0 hr and 6 hrs. (**D**) Binding of PBN to the oxidized βH. β-hematin was incubated with the different concentration of PBN (0–600 µM) in the presence of MetHb (7.75 µM), H_2_O_2_ and optical spectra were recorded.

Methemoglobin in the presence of H_2_O_2_ oxidizes a number of aromatic and halide substrates through single electron oxidation mechanism [37], [38], [39]. When H_2_O_2_ was added to native MetHb (Fe-III), a shift of soret peak from 406 nm (Figure 5A, spectrum a) to 417 nm (Figure 5A, spectrum b) was observed indicating the formation of an intermediate higher oxidation ferryl state (Fe^IV = O^) complex, compound II. When β-hematin (10 µM) was added to this complex (417 nm), it gets reduced to the native FeIII state (403 nm) by one electron transfer process (Figure 5A, spectrum c). An equal amount of β-hematin was added to the reference cuvette to avoid artifacts of shift in soret peak. The UV-Visible spectral studies strongly support that the β-hematin probably been oxidized to single electron containing species βH* following a single electron oxidation mechanism.

### Single electron containing species (βH*) is responsible for MetHb mediated β-hematin toxicity

Different concentration of βH in the presence of MetHb (7.75 µM) gives a dose-dependent killing of macrophages, if remains untreated. Whereas, cells incubated with the different concentration (50–300 µM) of PBN showed a dose-dependent recovery from the cytotoxic effect of β-hematin/MetHb with full recovery at 250 µM (Figure 5B). A light microscopic observation of PBN pre-incubated macrophages showed a full recovery from cellular damage. At the initial time point (0 hr), all cells exhibit normal phenotype but at 6 hr, PBN pre-incubated macrophages showed recovery from the toxicological phenotype of β-hematin/MetHb (Figure 5C). We further explored whether PBN is binding to the βH* and protecting macrophages from cytotoxic effects. PBN has a characterstic optical spectra with a λ_max_ at 288 nm in methanol [56]. β-hematin was incubated with the different concentration of (PBN 0–600 µM) in the presence of MetHb (7.75 µM), H_2_O_2_ and optical spectra was recorded. The optical spectra of β-hematin recovered from the reaction mixture has a characterstic peak at 287 nm and the height of the peak is proportional to the concentration of PBN (0–600 µM) present in the reaction mixture (Figure 5D). The presence of PBN in the β-hematin fraction indicates the formation of β-hematin-PBN complex with a stoichometery of 1∶1 in the lower concentration and 1∶2 in the higher concentration but the site of conjugation is not determined. The optical spectra of isolated βH in the absence of either MetHb or H_2_O_2_ is not giving a characteristic peak of PBN at 287 nm (data not shown). We have tried to identify the single electron species (βH*) in an EPR experiment, but high molecular weight and least solubility of βH in aqueous buffer system did not allow us to perform EPR spectroscopy. Incubating βH (60 µg/ml) in a peroxidase assay system (containing methemoglobin, H_2_O_2_) for 60 min at 37°C and macrophages treated with βH recovered from peroxidase reaction mixture for 6 hr at 37°C gives comparable level of toxicity as the macrophage treated with βH/MetHb (data not shown). Thus, the above result confirms that generation of a single electron species via one electron transfer from methemoglobin is responsible for the enhanced cyto-toxicity of βH to the macrophages.

### Macrophages follow an oxidative stress dependent apoptotic pathway in response to βH/MetHb exposure

RIP1 kinase is down-stream to stress linked signaling and crucial for cell’s fate to live or die. RIP1 kinase knockout animals exhibit enhanced apoptosis and death. Exposure of macrophages with hemin causes programmed necrosis through autocrine TNF and development of oxidative stress [57]. Piceatanol inhibits TNF induced NF-κB activation and down-stream gene expression [58]. To understand the role of TNF mediated autocrine signaling in this process, macrophages treated with combination of βH (60 µg/ml)/MetHb (7.75 µM) for 6 hr in the absence or presence of piceatanol (25 µM) and cellular viability was measured by MTT assay. Macrophage treated with combination of βH (60 µg/ml)/MetHb (7.75 µM) for 6 hr exhibits reduction in percentage cellular viability (45.23±0.54), whereas the percentage viability was partially restored in the presence of piceatanol (87.86±1.89). The cytotoxic effect of βH/MetHb is also restored in the presence of serum with percentage viability of 91.85±5.52. To further understand the role of TNF-α secreted from the macrophages, cells were treated with combination of βH (60 µg/ml)/MetHb (7.75 µM) for 6 hr in the presence of control antibodies (isotype IgG) or anti-TNF-α antibodies (20 µg for each reaction). The cells in presence of control antibody exhibits cellular viability (51.38±1.61) whereas the percentage viability was partially restored in the presence of anti-TNF-α antibody (93.13±2.75).

**Figure 6:**
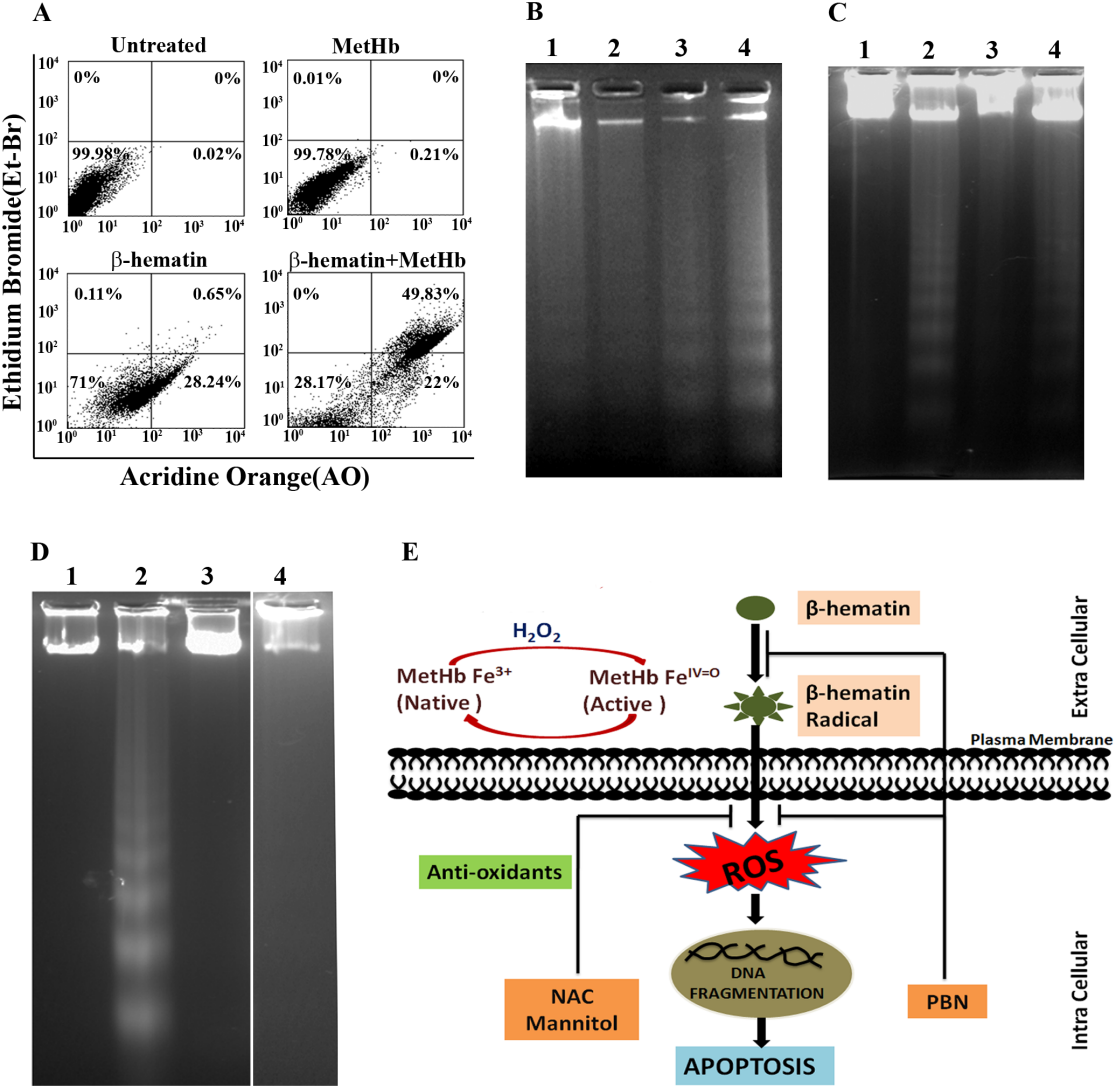
Macrophages follow oxidative stress mediated apoptosis to exhibit death. (**A**) Macrophages either remained untreated or treated with MetHb (7.75 µM), β-Hematin (60 µg/ml), or combination of β-Hematin (60 µg/ml)/MetHb (7.75 µM) for 6 hrs at 37°C in incomplete media. Cells were stained with acridine orange/Et-Br and analyzed in flow cytometry to characterize healthy, early or late apoptotic, and necrotic cell population. (**B**) DNA fragmentation analysis of macrophages treated with methemoglobin (7.75 µM), different concentration of β-Hematin (40 or 60 µg/ml) or combination of β-hematin (60 µg/ml)/methemoglobin (7.75 µM) in incomplete media for 6 hrs at 37°C. DNA fragmentation analysis was performed as described in “material and methods”. (**C**) Oxidative stress is essential for induction of apoptosis during treatment of macrophages with combination of β-hematin/methemoglobin. DNA fragmentation analysis of macrophages either remains untreated or treated with combination of β-hematin (60 µg/ml)/methemoglobin (7.75 µM) for 6 hrs at 37°C in the absence or presence of different antioxidant molecules (NAC/mannitol). DNA fragmentation analysis was performed as described in “material and methods”. (**D**) Methemoglobin mediated generation of βH* in the presence of extracellular H_2_O_2_ is responsible for macrophage apoptosis. DNA fragmentation analysis of macrophage remains untreated or treated with combination of β-hematin (60 µg/ml)/methemoglobin (7.75 µM) for 6 hrs at 37°C in absence or presence of catalase (200 units) or PBN (300 µM) respectively. DNA fragmentation analysis was performed as described in “material and methods”. (**E**) Schematic Diagram of methemoglobin mediated β-hematin toxicological potentiation towards macrophages. A details description is given in the text.

The differential population of apoptotic cells (early or late), necrotic and dead cells were identified by acridine orange-ethidium bromide (AO-EtBr) double staining method [44]. The Acridine Orange (AO) stains cells with early or late stage of apoptosisto give green fluorescence whereas ethidium bromide (Et-Br) binds to the DNA of necrotic/dead cells to give orange fluorescence. Macrophage treated with incomplete media (control), MetHb (7.75 µM), βH (60 µg/ml) or combination of βH/MetHb for 6 hr, and AO-EtBr double stained cells were analyzed by flow cytometry. Untreated or MetHb treated J774A.1 cells show 100% healthy cells (Figure 6A) where as β-hematin treated cells show 71.8% healthy, 28.2% apoptotic cells (Figure 6A, β-hematin). In the presence of methemoglobin, β-hematin treated cells exhibit 28.17% healthy, 22% early apoptotic and 49.83% late apoptotic/necrotic cells (Figure 6A, β-hematin/MetHb).

DNA fragmentation is used to assess the degree of apoptosis in macrophage cells [38]. Macrophages were treated with different concentration of βH (0–60 µg/ml), MetHb (7.75 µM) or combination of βH (60 µg/ml)/MetHb (7.75 µM) for 6 hr, genomic DNA was extracted from treated cells, and analyzed on 1.5% agarose gel. MetHb treated cells show an intact genomic DNA with very little or no visible appearance of DNA fragments (Figure 6B, lane 1). In contrast, cells treated with different concentration of βH give the appearance of DNA fragments (Figure 6A, lane 2, 3) whereas the level is significantly high in combination of βH (60 µg/ml)/MetHb (7.75 µM) treatment (Figure 6A, lane 4). DNA fragmentation pattern appeared in treated cells was completely reversed in the presence of antioxidants (Figure 6C). Removal of external hydrogen peroxide (by catalase) restored the integrity of the genomic DNA (Figure 6D, lane 3). In addition, scavenging of single electron containing species (such as βH*) by PBN also reversed the DNA fragmentation pattern confirmed the role of β-hematin radical (βH*) (Figure 6D, lane 4). Hence, data concludes the appearance of βH* (due to mutual interaction of β-hematin with MetHb in the presence of hydrogen peroxide) to induce DNA fragmentation (apoptosis) in an oxidative stress dependent manner. Caspases are down-stream to the death-receptor apoptotic signal. Active caspases degrades host proteins to execute the death program. To understand the role of caspases in this process, macrophages were treated with combination of βH (60 µg/ml)/MetHb (7.75 µM) for 6 hrs in the absence or presence of pan-caspase inhibitor z-VAD, genomic DNA was extracted from treated cells, and analyzed on 1.5% agarose gel. In the presence of z-VAD disappearance of DNA fragmentation is observed but partial DNA damage still present (Figure S1). In addition, macrophages treated with combination of βH (60 µg/ml)/MetHb (7.75 µM) for 6 hr in the absence or presence of pan-caspase inhibitor z-VAD, the cytotoxic effect of βH/MetHb is restored with percentage viability of 86.12±3.21. A further study is required to unreveal molecular events connecting oxidative stress to the down-stream effector components involved in the induction of apoptosis.

## Discussion

Propogation of malaria parasite within host blood releases a mixture of different pro-oxidant molecules such as methemoglobin, heme, haemozoin (heme polymer) and malaria toxins [5], [6], [7], [30], [47], [48]. Haemozoin is an inert non-biodegradable polymer and macrophages treated with polymer exhibit significant changes in cytokine profile (pro-inflammaory/anti-inflammatory), phagosome maturation, and other functional defects [16], [17], [18], [19], [20], [59]. Macrophages J774A.1 exposed to hemin and heme polymer exhibit significant change in cellular viability (Table 1). In the current study, heme polymer and βH exposure to macrophages cause dose-dependent reduction in cellular viability with LC_50_ of 132 µg/ml and 182 µg/ml respectively (Figure 1). In an in-vitro incubation system, methemoglobin accepts hemin as substrate and polymerizes to the heme polymer, and may further contributes into the level of toxic heme polymer [38]. In the presence of extracellular methemoglobin, βH toxicity towards macrophages is increased several folds with LC_50_ of 58 µg/ml (Figure 1). Pro-oxidant molecules released during malaria have ability to potentiate macrophage phagocytosis [49], [50] and may explain higher uptake of βH in macrophage treated with βH/MetHb (Figure 1). Methemoglobin exposure to the macrophages cause production of multiple ROS spikes within cytosol to develop oxidative stress [36]. Development of oxidative stress activates stress linked signaling components to secrete pro-inflammatory cytokines [13]. Macrophages exhibit higher level of phagocytosis in the presence of pro-inflammaotory cytokineses [49], [50]. Hence, a series of molecular events down-stream to methemoglobin mediated ROS production may in-turn activates macrophage machinery to stimulate higher uptake of βH through phagocytosis. Macrophages isolated from malaria patients exhibit different degree of phagocytotic activity, accumulation of intracellular ROS, and functional defects within macrophages correlate well with the degree of phagocytotic activity. A detail study may be needed to understand molecular events responsible for higher phagocytotic activity and resulting accumulation of βH within macrophages.

Peroxidases play a pivotal role in protecting cells from generated peroxide and other free radicals. Methemoglobin has pseudoperoxidase activity and it has potential to oxidize aromatic and halide substrates [37], [38], [39]. Dual role of methemoglobin in cyto-protective or cytotoxic effect is documented and it is linked to the availability and ability of methemoglobin to oxidize particular substrate [60]. Substrate oxidation through peroxidases utilizes large amount of peroxide to form stable product, and protect cells from high level of peroxides. In cases where peroxidase is incompetent to oxidize the substrate or substrate oxidation product is fast acting (unstable), results in enhanced cytotoxicity [8]. Methemoglobin accepts primaquine as substrate and oxidizes it to the very fast acting 5-hydroxy-primaquine and enhances the hemo-toxicity of parent molecule towards RBCs [37]. Following similar mechanism, methemoglobin utilizes extracellular H_2_O_2_ to oxidize βH to generate βH* (Figure 4). It is interesting that extracellular H_2_O_2_ is important for enhanced toxicity of βH in the presence of methemoglobin (Figure 3). Methemoglobin to hemoglobin turn over through oxidation/reduction reactions (by several pro-oxidant/antioxidant molecules) are releasing free electron into the aqueous environment and might be responsible for generation of superoxide due to reactions of methemoglobin with H_2_O_2_ [8]. PBN mediated reversal of cytotoxic effects of βH partially supports such a mechanism but does not rule out the obvious role of βH* radical as well (Figure 4). A schematic model to summarize the current finding is presented in Figure 6. Methemoglobin mediated toxicological potentiation of pro-oxidant β-hematin towards macrophages involve multiple steps: **Step 1**; Methemoglobin oxidizes βH to form βH* in the presence of excess H_2_O_2_ in extracellular milieu. Scavenging of H_2_O_2_ with catalase reduces the cytotoxic potential of βH*indirectly supports that βH* radical generated in the cell free system is responsible for enhanced cytotoxicity. Simultaneously, it is potentiating macrophage phagocytotic activity to engulf large amout of βH crystals. **Step 2**; Irrespective of β-hematin uptake mechanism, βH accumulation inside the macrophages cause development of oxidative stress. The most probable site of βH accumulation may be macrophage phagosomal system to causes induction of apoptosis mediated macrophage death. A large number of evidences support the model but further studies are required to decipher underlying molecular events.

## Supporting Information

Figure S1 **Caspase activity is responsible for observed Apoptosis in macrophage. DNA fragmentation analysis.** DNA fragmentation analysis of macrophages either remains untreated or treated with combination of β-hematin (60 µg/ml)/methemoglobin (7.75 µM) for 6 hrs at 37°C in the absence or presence of pan caspase inhibitor z-VAD. 1 = untreated, 2 = macrophage treated with combination of β-hematin (60 µg/ml)/methemoglobin (7.75 µM) and 3 = 2, in the presence of z-VAD. DNA fragmentation analysis was performed as described in “material and methods”.
(TIF)
