# Supplementary figures and images for: Phagocytic Uptake of Oxidized Heme Polymer Is Highly Cytotoxic to Macrophages
Source: PLoS One. 2014 Jul 31;9(7):e103706. doi: 10.1371/journal.pone.0103706 (PMC4117526; doi:10.1371/journal.pone.0103706)

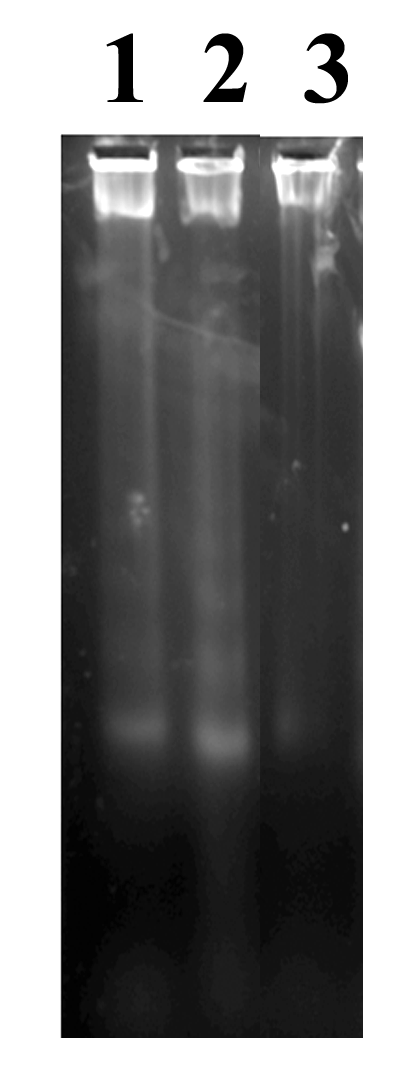

Supplement: Figure S1 — Caspase activity is responsible for observed Apoptosis in macrophage. DNA fragmentation analysis. DNA fragmentation analysis of macrophages either remains untreated or treated with combination of β-hematin (60 µg/ml)/methemoglobin (7.75 µM) for 6 hrs at 37°C in the absence or presence of pan caspase inhibitor z-VAD. 1 = untreated, 2 = macrophage treated with combination of β-hematin (60 µg/ml)/methemoglobin (7.75 µM) and 3 = 2, in the presence of z-VAD. DNA fragmentation analysis was performed as described in “material and methods”. (TIF) [file pone.0103706.s001.tif]
